
JOURNAL INFORMATION
==============================
NLM Title Abbreviation: Pediatr Rheumatol Online J
Journal ID: Pediatr Rheumatol Online J
NLM Journal ID: 101248897

Pediatric Rheumatology Online Journal

EISSN: 1546-0096
Publisher: BMC

ARTICLE INFORMATION
==============================
PMCID: PMC7725540
DOI: 10.1186/s12969-020-00478-x
Article ID: 478
Article version: 2
Subjects: Meeting Abstracts

Proceedings of the 26th European Paediatric Rheumatology Congress (PReS 2020) - Late Breaking Abstracts

Virtual. 23 - 25 September 2020

Electronic publication date: 2020 Dec 10
Publication date: 2020
Volume: 18
Issue: Suppl 3
Issue sponsor: Publication charges for this supplement were provided by the Paediatric Rheumatology European Society
Electronic Location ID: 93
Copyright: © The Author(s) 2020
License: Open AccessThis article is licensed under a Creative Commons Attribution 4.0 International License, which permits use, sharing, adaptation, distribution and reproduction in any medium or format, as long as you give appropriate credit to the original author(s) and the source, provide a link to the Creative Commons licence, and indicate if changes were made. The images or other third party material in this article are included in the article's Creative Commons licence, unless indicated otherwise in a credit line to the material. If material is not included in the article's Creative Commons licence and your intended use is not permitted by statutory regulation or exceeds the permitted use, you will need to obtain permission directly from the copyright holder. To view a copy of this licence, visit http://creativecommons.org/licenses/by/4.0/. The Creative Commons Public Domain Dedication waiver (http://creativecommons.org/publicdomain/zero/1.0/) applies to the data made available in this article, unless otherwise stated in a credit line to the data.
License URL: https://creativecommons.org/licenses/by/4.0/

Conference name: 26th European Paediatric Rheumatology Congress (PReS 2020)
Conference location: Virtual
Conference date: 23 - 25 September 2020
issue-copyright-statement: © The Author(s) 2020

==============================
Download to read this full article text.

Publisher’s Note

Springer Nature remains neutral with regard to jurisdictional claims in published maps and institutional affiliations.

